# Percutaneous transhepatic approach to endoscopic placement of a 10F plastic biliary stent: step-by-step description of a novel technique

**DOI:** 10.1590/0100-3984.2019.0116

**Published:** 2020

**Authors:** Thiago Franchi Nunes, Rômulo Florêncio Tristão Santos, Tiago Kojun Tibana, Denis Szejnfeld

**Affiliations:** 1 Hospital Universitário Maria Aparecida Pedrossian da Universidade Federal de Mato Grosso do Sul (HUMAP-UFMS), Campo Grande, MS, Brazil.; 2 Escola Paulista de Medicina da Universidade Federal de São Paulo (EPM-Unifesp), São Paulo, SP, Brazil.

## INTRODUCTION

The endoscopic placement of plastic biliary stents is a well-established technique for treating benign, malignant, or recurrent biliary obstructive disease^(1,2)^. Although the percutaneous transhepatic technique is a well-known means of inserting external drains and metallic biliary stents^(1,3-7)^, there has been only one study providing a technical description of the percutaneous transhepatic insertion of a plastic biliary stent, and the authors of that study employed a two-stage approach^(1)^. To our knowledge, there have been no articles describing the technique in a single-stage approach and using a 10F biliary stent.

## PROCEDURE

Preoperative images should be reviewed in detail for anatomical definition, possible anatomical variants, location of the point of obstruction, determination of the best positioning of the patient, and planning of the puncture site (the right or left hepatic duct).

**Figure 1:**
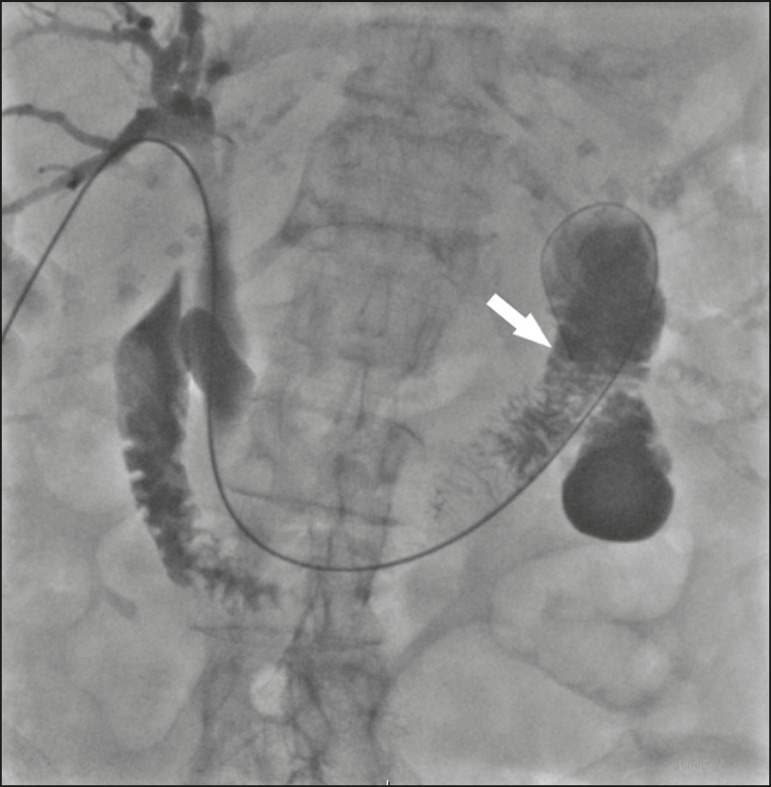
A 0.035” hydrophilic guidewire and a 5F diagnostic catheter with its distal end positioned inside the duodenum, near the ligament of Treitz (arrow).

With the patient under conscious sedation, local anesthesia (10 mL of 2% lidocaine) is administered at the point of percutaneous puncture. Percutaneous transhepatic puncture is then performed with a 17G × 10.6 cm coaxial needle (MCXS1816AX; Argon Medical Devices, Frisco, TX, USA) and a 5F vascular introducer (RS+A50K10SQ Radifocus Introducer II; Terumo, Tokyo, Japan), guided by ultrasound, through a peripheral branch of the bile duct. That is followed by cholangiography to visualize the exact location of the obstruction. A 0.035” hydrophilic guidewire and a 5F vertebral diagnostic catheter (IMPULSE; Boston Scientific, Marlborough, MA, USA) are then inserted, past the point of obstruction, until the distal end of the catheter is positioned inside the duodenum, preferably next to the ligament of Treitz (Figure 1). The entire duodenal lumen is then distended with approximately 25 mL of the iodinated contrast medium iodixanol (Visipaque; GE Healthcare, Chicago, IL, USA) diluted in 100 mL of 0.9% saline solution (total volume, 125 mL), for better visualization of the duodenal lumen and for planning the insertion of the 10F × 12 cm plastic stent, previously assembled with the delivery system (Percuflex; Boston Scientific) to avoid improper positioning and iatrogenic lesions of the duodenal wall by the stent. The hydrophilic guidewire is replaced by a 0.035” rigid guidewire (Amplatz Super Stiff; Boston Scientific), and the diagnostic catheter is removed. Then the 5F introducer is replaced by an 11F vascular introducer (RS*A11K10SQ Radifocus Introducer II; Terumo) to feed in the 10F × 12 cm plastic stent.

**Figure 2:**
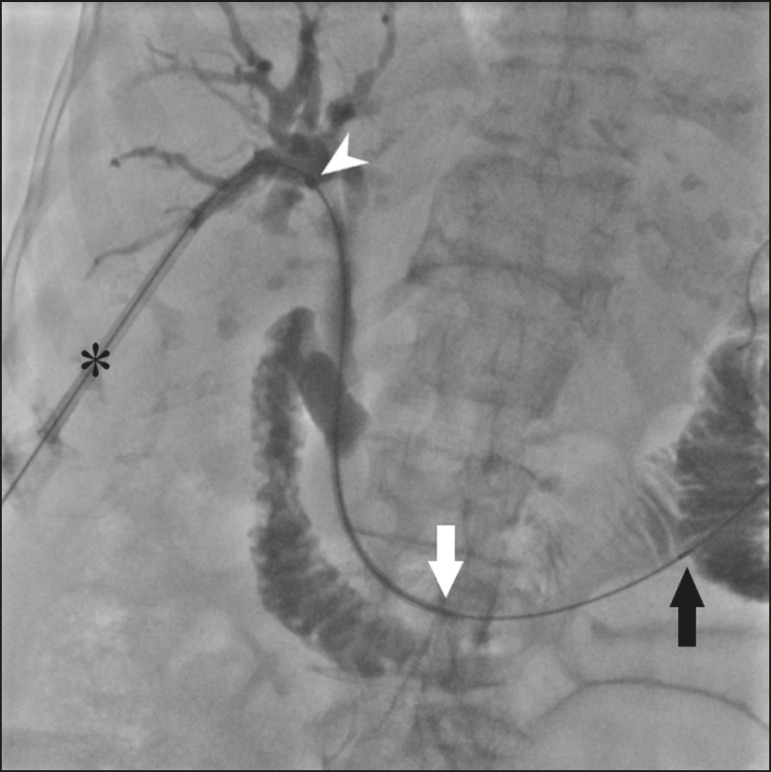
An introducer is positioned (asterisk). The stent, together with the delivery system (white arrow), is inserted along the guidewire through the lumen of the introducer. The radiopaque mark on the delivery system (arrowhead) is located 1 cm above the proximal end of the plastic biliary stent, which will be positioned inside the bile duct (above the point of obstruction). Under fluoroscopy, we carefully visualize the radiopaque mark on the delivery system (black arrow), which delimits the distal portion of the plastic stent, and position it approximately 5 cm below its anchoring in the duodenum.

**Figure 3:**
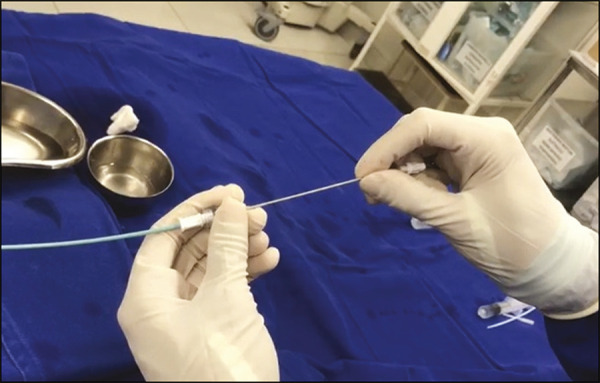
Photograph of the distal end of the plastic biliary stent delivery system.

**Figure 4:**
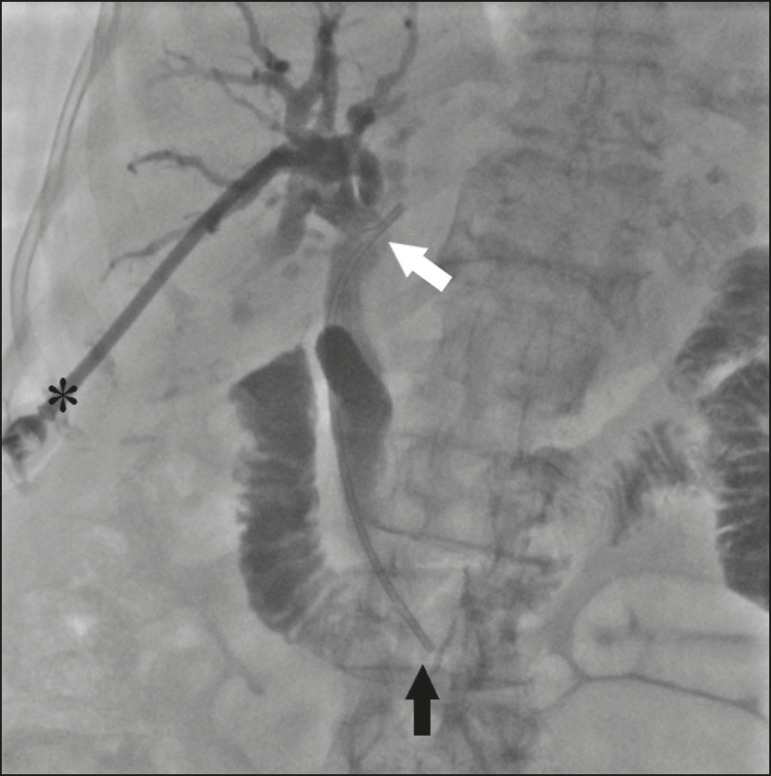
After removing the delivery system, we inject a small amount of diluted iodized contrast through the introducer (asterisk) and conduct a postprocedure cholangiography. That allows us to evaluate the patency of the stent, as well as its positioning, with its proximal end at the level of the common hepatic duct (white arrow) and its distal end at the level of the duodenum (black arrow).

The stent, together with the delivery system, is inserted over the guidewire through the sheath of the 11F introducer (Figure 2). Under fluoroscopy, we first seek to visualize and identify the two radiopaque marks, which are of utmost importance for the proper positioning of the plastic biliary stent. The distal radiopaque mark delimits one end of the delivery system at an estimated distance of 5 cm from the distal part of the stent (which will be inside the duodenum). The proximal radiopaque mark is 1.0 cm from the proximal portion of the plastic stent. The 11F introducer is pulled to a point at which the proximal portion of the stent is at an estimated distance of 3.0 cm above the bifurcation of the intrahepatic bile duct (in the direction of the puncture site), so as to correctly position that portion of the stent and confirm that it is not visible within the introducer. At this point, the rigid 0.035” guidewire is removed completely in order to improve the accommodation and anchoring of the distal portion of the stent inside the duodenum. The surgeon then releases the plastic stent by unscrewing the outer part of the delivery system (four turns) and removes it entirely (Figure 3). When the delivery system is being removed, the proper distal anchoring of the stent inside the duodenum and the proper positioning of its proximal end within the bile duct, should be confirmed by the radiopaque marking (Figure 4). After the stent delivery system has been removed, the stent can no longer be repositioned. As part of the protocol at our facility, a follow-up abdominal X-ray is taken 24 h after the procedure in order to verify that the intrabiliary contrast has been completely eliminated. In all cases of lesions suspected of malignancy, cholangiobiopsies are performed with the technique described by Nunes et al.^(6)^.

Percutaneous drainage via a plastic biliary stent is a technique that is easily performed, from a technical point of view. We believe it to be an extremely safe, affordable, and feasible option for the treatment of symptomatic obstructive jaundice in a variety of scenarios.
